# Statistical learning at a virtual cocktail party

**DOI:** 10.3758/s13423-023-02384-1

**Published:** 2023-10-02

**Authors:** Christina Papoutsi, Eleni Zimianiti, Hans Rutger Bosker, Rebecca L. A. Frost

**Affiliations:** 1 Max Planck Institute for Psycholinguistics, PO Box 9104, 6500 HE Nijmegen, The Netherlands; 2 Donders Institute for Brain, Cognition, and Behaviour, Radboud University, Nijmegen, the Netherlands; 3 Edge Hill University, Edge Hill, UK

**Keywords:** Auditory statistical learning, Cocktail party listening, Speech perception, Selective attention, Transitional probabilities

## Abstract

Statistical learning – the ability to extract distributional regularities from input – is suggested to be key to language acquisition. Yet, evidence for the human capacity for statistical learning comes mainly from studies conducted in carefully controlled settings without auditory distraction. While such conditions permit careful examination of learning, they do not reflect the naturalistic language learning experience, which is replete with auditory distraction – including competing talkers. Here, we examine how statistical language learning proceeds in a *virtual cocktail party environment*, where the to-be-learned input is presented alongside a competing speech stream with its own distributional regularities. During exposure, participants in the Dual Talker group concurrently heard two novel languages, one produced by a female talker and one by a male talker, with each talker virtually positioned at opposite sides of the listener (left/right) using binaural acoustic manipulations. Selective attention was manipulated by instructing participants to attend to only one of the two talkers. At test, participants were asked to distinguish words from part-words for both the attended and the unattended languages. Results indicated that participants’ accuracy was significantly higher for trials from the attended vs. unattended language. Further, the performance of this Dual Talker group was no different compared to a control group who heard only one language from a single talker (Single Talker group). We thus conclude that statistical learning is modulated by selective attention, being relatively robust against the additional cognitive load provided by competing speech, emphasizing its efficiency in naturalistic language learning situations.

**Supplementary Information:**

The online version contains supplementary material available at 10.3758/s13423-023-02384-1.

## Introduction

To achieve linguistic proficiency, learners must develop the ability to parse incoming speech into individual words. While there are no perfectly reliable cues for word boundaries in spoken language (Aslin et al., 1996; Liberman & Studdert-Kennedy, 1978), there are a myriad of sources of information that learners draw upon to segment speech (e.g., Stärk et al., 2021) – including distributional information. Transitional probabilities (TPs) between syllables inform listeners about what may constitute a word: TPs between syllables within words are typically higher than TPs for syllables spanning word-boundaries, providing a helpful indication of where words may begin and end (e.g., Saffran et al., 1996b; Turk-Browne et al., 2008).

The ability to draw upon TPs (*statistical learning*) has been found to aid word segmentation in learners of all ages (Aslin et al., 1998; Saffran et al., 1996a, 1997), giving rise to the suggestion that statistical learning (SL) may play a key role in language acquisition (see e.g., Conway et al., 2010; Frost et al., 2020; Kidd & Arciuli, 2016). Although most research providing evidence for human’s SL ability used artificial languages as stimuli, this finding has been replicated using natural language too (Pelucchi et al., 2009). SL research with more ‘naturalistic’ artificial language input (i.e., reflecting the distributional properties of TPs in natural language) has documented profound learning advantages when the to-be-learned material aligns with participants’ prior knowledge of TPs (Elazar et al., 2022; Stärk et al., 2022). Yet, critically, most evidence for SL predominantly stems from studies examining learning in a ‘vacuum’, under strict laboratory conditions, and without distraction. However, language acquisition typically proceeds amid a plethora of speech-intrinsic (e.g., between-talker pronunciation variation; Estes & Lew-Williams, 2015) and speech-extrinsic noise, such as background sounds and competing speech signals. Such variability can prove challenging and may critically impact learning (e.g., Benitez et al., 2020; Samuel, 2016; Zeamer & Fox Tree, 2013). Here, we examine how SL proceeds in the presence of auditory distractions, comparing speech segmentation and word recognition of a to-be-attended input source presented concurrently with a competing speech stream with its own distributional regularities. Demonstrating that SL can proceed in the presence of competing linguistic input is critical for shaping our understanding of the role of SL in natural language acquisition.

Learners must cope with ‘noise’ in many communicative situations. Humans are highly adept at segregating multiple auditory signals in multi-talker environments (i.e., ‘cocktail party’ settings) (Bosker et al., 2020a; Bronkhorst, 2000; McDermott, 2009). This is accomplished by selectively attending to the speech source of interest while filtering out others. However, this is indisputably challenging, depleting resources as a result of *cognitive load*, typically reducing performance on attended speech in multi-talker listening conditions compared to in quiet (Mattys et al., 2012). Furthermore, *selective attention* is not perfect: some acoustic and linguistic properties of to-be-ignored speech persistently influence the processing of attended speech (e.g., speech rate, Bosker et al., 2017, 2020b; linguistic informational masking, Dai et al., 2017). This raises the question how SL of distributional regularities in attended speech operates in multi-talker contexts. Specifically, does cognitive load reduce SL of attended speech in ‘cocktail party’ contexts compared to in quiet? Also, how successful are listeners in ignoring the distributional regularities in to-be-ignored speech?

Some studies using dual-tasking paradigms suggest that auditory SL is reduced as cognitive demands on attentional resources increase (Palmer & Mattys, 2016; Toro et al., 2005, 2011). However, others claim that auditory SL remains unaffected (Batterink & Paller, 2019; Daikoku & Yumoto, 2017, 2019), or is only partially affected, by an increase in cognitive demands (Fernandes et al., 2010). The inconsistency in results could be due to variation in the modality and nature of the distractor stimuli (e.g., visual vs. auditory; speech vs. non-speech) and their similarity to target stimuli (Conway & Christiansen, 2006). Similar mixed findings regarding cognitive load and selective attention are present in SL in the visual modality (e.g., Campbell et al., 2012; Musz et al., 2015; Turk-Browne et al., 2005). To date, no study has assessed auditory SL in concurrent multi-talker contexts (i.e., with simultaneously speaking talkers; for sequential multi-talker settings, see, e.g., Benitez et al., 2020), which could prove particularly challenging since the target and distractor are similar in nature.

Literature on how SL operates on unattended input is scarce. SL does not require active processing; humans learn TPs even when passively listening (Saffran et al., 1997). However, to our knowledge, only two studies tested to what extent unattended TPs are learned when instructed to deliberately attend another input stream in the same (auditory) modality. Daikoku and Yumoto (2017, 2019) presented listeners with two concurrent tone streams and found evidence that participants performed above chance on both the attended and unattended streams. However, the possible effect of increased cognitive load due to concurrent exposure to multiple tone sequences relative to a single stream was not tested. Crucially, it remains unclear whether similar findings would be observed when listening to (acoustically more complex) speech stimuli in multi-talker contexts.

The present study mimicked a ‘cocktail party’ setting using a dichotic listening paradigm, where participants concurrently heard two novel language streams produced by two different talkers (Dual Talker group), perceived as coming from opposite sides. Each language was made up of unique syllables and hence involved unique TPs (e.g., Language A: *zutami pejuxo nisuda…*; Language B: *pomasi vukoza fanujo…*). During exposure, participants were instructed to attend to only one talker while ignoring the other. Participants were subsequently tested on (i) ‘segmentation trials’ assessing their ability to identify words versus part-words from the attended (*nisuda* vs. *xo#nisu*) and unattended language (*fanujo* vs. *za#fanu*) and (ii) ‘recognition trials’ assessing their discrimination of words from the attended language versus words from the unattended language (*nisuda* vs. *fanujo*). In addition, a control group (Single Talker group) was presented with only one talker, allowing comparison of SL of a given language in competing speech versus quiet. If SL is modulated by the cognitive load of having to selectively attend one talker while ignoring another, participants in the Dual Talker group should show lower accuracy on segmentation trials of the attended language compared to the Single Talker group (i.e., distinguishing words from part-words) as well as worse performance on recognition trials (discriminating a word from the attended vs. unattended language). Moreover, if SL is modulated by selective attention, participants in the Dual Talker group should demonstrate higher accuracy on segmentation trials from the attended versus unattended language.

## Method

### Participants

Participants were 96 adult native speakers of Dutch (age: *M* = 27 years, range = 18–40 years; 38 females and 42 males), all of whom were recruited through the Prolific database (https://www.prolific.co), receiving monetary compensation for their time. The age limit of 40 years was set for two reasons: (1) as an analogy to corresponding work employing university samples, and (2) with the aim to avoid the recruitment of participants with possible reduced hearing acuity. Data from one participant were excluded due to technical issues, leaving data from 95 participants for analysis. None of the participants reported any auditory, speech, language, or attention deficit (e.g., ADHD) disorders. Prior to their participation in the online experiment, all subjects provided consent after being thoroughly informed about the study at hand, following the guidelines approved by the Ethics Committee of the Social Sciences department of Radboud University (project code: ECSW-2019-019).

### Design

The experiment adopted a typical SL paradigm including a familiarization phase and a subsequent test phase. We used a between-participants design involving two groups (*Single Talker* and *Dual Talker*; randomly assigned), who received different familiarization phases but identical test phases. The Single Talker group heard one talker producing a single ‘Language’, mirroring the vast majority of SL studies. The Dual Talker group was presented with two simultaneously speaking talkers, each producing a different language, while being instructed to attend to only one of them.

### Materials

#### Stimuli

Two artificial languages were created (*Language A* and *Language B*), each containing six unique three-syllable novel words (see Table 1). Both languages consisted of the same 18 consonants (p, b, t, d, k, f, v, s, z, ʃ, x, h, m, n, ʋ, l, j, r) and five vowels (a, e, i, o, u). The consonants and vowels were then pseudorandomly combined into 18 CV syllables per language, with each consonant occurring only once in each language, and each vowel occurring between three and four times. All CV combinations formed phonotactically legal syllables in Dutch. The syllables were unique both within and between the two languages (e.g., /xo/ in Language A, but /xi/ in Language B). We then pseudorandomly combined the syllables into six trisyllabic words per language, such that (1) consonant manner of articulation (e.g., stops) and vowels (e.g., /e/) occurred equally in all three syllable positions, (2) they did not contain any existing Dutch multisyllabic words, and (3) no two words comprised the same vowels or consonants in the same order.

**Table 1:** Experimental words and part-words for each language

Language A		Language B	
words	part-words	words	part-words
/nisuda/	/xo # nisu/	/fanujo/	/za # fanu/
/pejuxo/	/mi # peju/	/vukoza/	/ʃa # vuko/
/zutami/	/lu # zuta/	/heriʃa/	/tu # heri/
/bavolu/	/volu # ho/	/pomasi/	/masi # he/
/hoʃife/	/ʃife # ni/	/biʋetu/	/ʋetu # xi/
/kireʋa/	/reʋa # ni/	/xidule/	/dule # po/

# indicates a word boundary

The stimuli were created from isolated syllable recordings of a female and male native speaker of Dutch who were instructed to speak in a monotone voice. The syllables were then processed using PSOLA in *Praat* (Boersma & Weenink, 2021) to have a fixed fundamental frequency (F0) of 190 Hz for the female talker and 130 Hz for the male talker (i.e., monotone speech at each talker’s average F0). The duration of each syllable was scaled to 300 ms, and the intensity was normalized to 70 dB. Our speakers happened to produce relatively long fricatives that disrupted the perceived isochrony of the concatenated syllable streams. Therefore, the fricatives were first slightly reduced in duration before scaling the entire syllable to 300 ms. Finally, each trisyllabic word was created by concatenating the three relevant syllables, such that each word was 900 ms long.

To create the input streams for the familiarization phase, we used Python (Kluyver et al., 2016) to randomly combine the six words of each Language into an orthographic sequence, avoiding immediate repetitions of individual words. These orthographic sequences then in turn served as the input to a Praat script that concatenated the auditory words into a stream. For each stream, within-word transitional probabilities were always 1.0, while the between-word transitional probabilities were on average 0.2 (range = 0.13–0.27). We created speech streams for each talker (male and female) for each of the two languages (A and B), resulting in four speech sequences (2 languages × 2 talkers).

All auditory streams were 10 min long and were continuous, with no pauses within or between words. Streams had a 5-s linear fade in and fade out to avoid cuing particular word onsets. To ensure participants’ attention throughout the duration of the familiarization stream, they were required to perform a simple beep detection task while listening to the language. Twelve beeps (each 100 ms; frequency = 440 Hz; intensity = 95 dB) were added to the streams at pseudorandom temporal positions, such that (1) they were counterbalanced across syllable positions (i.e., four beeps per syllable position within words), (2) they always occurred 100 ms after syllable onset to avoid energetic masking of an entire consonant or vowel of the syllable, (3) one beep occurred every 50 s, with the minimal temporal distance between beeps being greater than 15 s. Beeps were always played diotically in both ears.

### Procedure

Each experimental session consisted of the following procedure (same order across participants): first, participants provided informed consent after having been informed about the nature of the study. Following this, they performed two headphone screening tests which determined their eligibility to participate in the experiment proper. Then, they completed the familiarization and testing phases. Finally, participants filled out a post-experimental questionnaire.

#### Headphone screening tests

Psytoolkit (Stoet, 2010, 2017) was used to program and host the experiment online. Participants were instructed to use headphones and to complete the experiment in a quiet environment without any distractions. Participants first performed two headphone screening tests: The first aimed to ensure that participants were indeed using headphones, and was based on Huggins’ pitch (an illusory pitch phenomenon that can only be observed with dichotic stimulus presentation; Milne et al., 2020). The second test aimed to ensure proper binaural sound localization. This was achieved by manipulating three binaurally presented white noise sounds in both interaural time difference (ITD) and interaural intensity difference (IID). These manipulations were identical to those applied to the dichotic familiarization streams; see below for details. With these manipulations, two of the three noise sounds were perceived as left-lateralized and one as right-lateralized. On six trials, participants were asked to indicate which noise sound was perceived as coming from the right (noise 1, 2, or 3). Participants were only able to continue the experiment if they passed five out of six trials in each screening test. If participants failed, they were given one more chance to redo that screening test. If they failed once more, they were excluded from the experiment. Only participants who passed these tests are reported in this paper.

#### Familiarization

Participants were presented with a 10-min familiarization stream. For the Single Talker group, familiarization included diotic exposure (i.e., heard equally in both ears) to one of the four 10-min long speech sequences (male Language A, male Language B, female Language A, female Language B), which were counterbalanced across participants. For the Dual Talker group, familiarization included concurrent exposure to two speech sequences (Language A and Language B), where one was produced by a female talker and the other by a male talker. Both talkers were presented to both ears, but we used binaural ITD and IID manipulations to allow for spatial segregation of the two talkers, such that one talker was perceived as talking from the right and the other as talking from the left (i.e., dichotic stimulus presentation inducing a ‘virtual auditory reality’). This was implemented by applying an interaural time difference (ITD) of 600 μs and an interaural intensity difference (IID) of 6 dB for each talker. In this way, we simulated fully lateralized sound sources (Hartmann, 1999) at an overall signal-to-noise ratio (SNR) of 0 dB (aggregating over the two channels). This technical setup was motivated by our aim to present relatively naturalistic spatial segregation cues, resembling the way in which people receive input from competing speech signals in a ‘cocktail party’ environment.

**Fig. 1:**
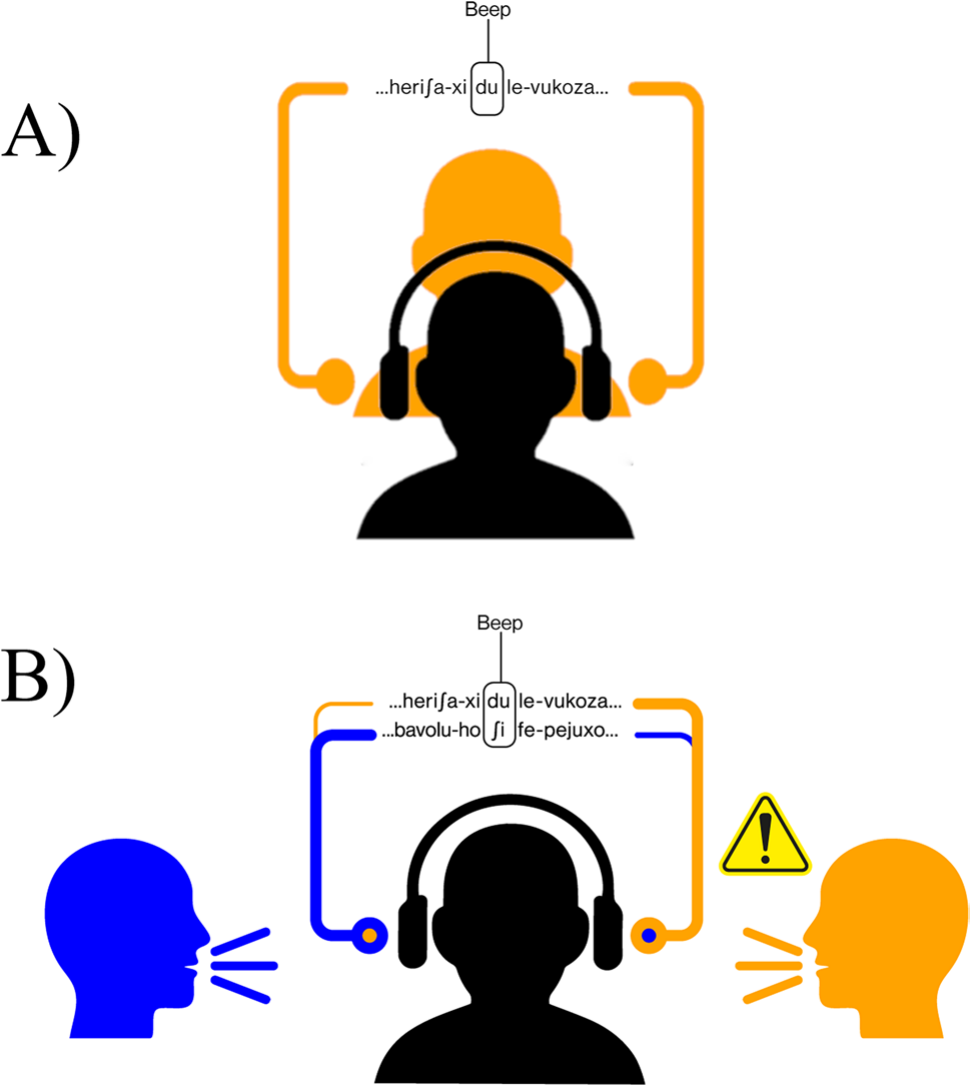
Example of Single Talker (**A**) and Dual Talker (**B**) stream presentation. The colored lines indicate the different speech streams produced by a male (blue) and female (orange) talker. Interaural differences of the two streams in (**B**) are displayed with line thickness, where a thicker line indicates a 600-μs lead and 6-dB greater intensity, leading to perceived spatial localization of the thicker line to the corresponding ear). Participants in the Single Talker group heard one talker equally in both ears (diotic presentation), whereas participants in the Dual Talker group heard two talkers such that one talker’s voice was perceived as coming from the right (orange head figure), and the other talker’s voice was perceived as coming from the left (blue head figure). Participants were instructed to pay close attention to one of the two talkers (here: the talker predominantly heard from the right). Listen to one of the dual talker streams at the following: https://osf.io/qn8a3, using headphones

Note that online studies of spoken language processing are best run using headphones (i.e., not speakers) to attenuate environmental noise. A simple way to present two lateralized speech signals over headphones would be to play one talker in the left ear and the other talker in the right ear. However, this kind of dichotic signal presentation over headphones underestimates the difficulty listeners face in everyday life, where the speech from any given talker typically reaches both ears, not just one. It also removes any energetic masking from one talker to the next because each talker is presented to one ear only. Moreover, such a simplistic design would allow participants in the Dual Talker group, who were tasked to only attend one talker and ignore the other, to take off one side of the headphones/earbuds (i.e., ‘cheating’). In real conversations, listeners rely on the binaural ITD/IID cues to successfully segregate two speech signals from spatially opposing talkers in order to overcome energetic masking. Therefore, in this experiment, we applied naturalistic ITD/IID cues at a ‘doable’ SNR of 0 dB (i.e., relatively good intelligibility for both streams) to closely resemble spatially segregated speech signals in a face-to-face conversation. Also, this removed the possibility of cheating (i.e., taking one side of the headphones off), because the speech from both talkers was always present in either ear (see Fig. 1). To get an impression of the virtual auditory reality, you can listen to one of the dual-talker streams here: https://osf.io/qn8a3 (using headphones).

The input streams from the two talkers were always presented in-phase: the syllable onset of one language always coincided with the syllable onset of the other. Exposure to each language + talker + side combination (e.g., Language A, produced by the male talker, perceived as coming from the left) was counterbalanced between participants, using four familiarization stimuli that were equally distributed across participants assigned to the Dual Talker group. An illustration of Single and Dual Talker stream presentation is displayed in Fig. 1. All stimuli are available in the Open Science Framework (OSF) repository (see *Data availability*).

Participants in the Single Talker group were instructed to pay close attention to the sequence they heard, and participants in the Dual Talker group were asked to attend to only one of the two speech sequences they were exposed to (counterbalanced across participants). Both groups were informed that they would later be tested on what they heard without explicitly describing the nature of the test. For the Dual Talker group, a reminder to focus on the stream they were assigned to was displayed on the screen with a message (e.g., *Listen carefully to the talker in your left ear)* and an arrow (e.g., pointing to the left) throughout familiarization. Participants in the Single Talker group were presented with a fixation point during familiarization. Participants in both groups completed the beep detection task during the familiarization phase, pressing the spacebar key on their computer keyboard as soon as possible when they heard a beep. An example of the beeping sound they were expected to identify was provided to the participants prior to familiarization. This beep detection task was included to motivate the participants to maintain auditory attention (i.e., not put down their headphones).

#### Testing

Participants completed a two-alternative forced-choice (2AFC) task consisting of 108 trials: 72 *segmentation trials*, half of which tested the attended language and half the unattended language, and 36 *recognition trials*. During either testing phase, participants were instructed to listen carefully to each test pair and select which item best matched the language they had just heard (for the Single Talker group) or paid close attention to (for the Dual Talker group), by pressing “A” for the first or “B” for the second sound stimulus on the computer keyboard. Participants had three seconds to respond after the second stimulus offset, after which there was a timeout and the next trial began.

Segmentation trials consisted of word versus part-word comparisons, where part-words were made up of three syllables that straddled a word boundary, comprising the last two syllables of one target word and the first syllable of another (type I, e.g., word: *kireʋa*; part-word: *reʋa#ni)* or the last syllable of a word and the first two syllables of a target word (type II, e.g., word: *nisuda*, part-word: *xo#nisu*; see Table 1). For each language (attended and unattended), the part-words were six in total, three of type I and three of type II. Each word was combined with each of the six part-words, resulting in 36 word + part-word combinations for each language.

Note that this design, whereby words and part-words occur equally often during the test phase, was motivated by an anonymous reviewer comment on an earlier experiment (referred to as Experiment S1). That earlier Experiment S1 mirrored the present study except for the critical difference that Experiment S1 presented each word twice during the test phase, each time paired with a different unique part-word. Thus, words occurred twice as often during the test phase compared to part-words, providing an opportunity for participants to ‘learn’ the words of a given language over the course of the test phase. The results of Experiment S1 were qualitatively similar to the ones reported here lending additional support to our present findings; for full description, see https://osf.io/zc543/. However, they also showed evidence of within-test learning which could be argued to dilute the clarity of the outcomes. Therefore, we ran a new experiment with a new participant sample, this time presenting each unique word and part-word equally often during test, thus removing any opportunity for within-test learning (i.e., reported here). Further distinctions between Experiment S1 and the present experiment are: (1) Experiment S1 had two versions of each Language (A and B) to control for item-specific biases, but results did not demonstrate any differences between versions and therefore this design aspect was dropped for the present experiment; (2) the recognition test phase in Experiment S1 had only 12 trials, while in the present design 36 recognition trials were presented (see below, in comparison to https://osf.io/zc543/).

On each segmentation trial, words and part-words were presented auditorily (diotically) with ISI = 1,000 ms. The order in which each word and part-word occurred as well as the talker producing both items (i.e., male and female) was counterbalanced across the segmentation trials. Note that, while words were heard in only one talker’s voice during familiarization (either male or female), they were produced by both talkers at test (half of the time by the ‘congruent’ talker (i.e., same talker as in familiarization); half of the time by the ‘incongruent’ talker. Thus, we aimed to avoid influences of episodic memory (e.g., word + talker combinations) on test performance.

After all 72 segmentation trials, participants were presented with 36 recognition trials. These consisted of test pairs made up of a word from Language A + a word from Language B (e.g., *nisuda* vs. *fanujo).* Each word from Language A was paired with every word from Language B, resulting in 36 trials in total. These recognition trials were included as they would illustrate the degree of familiarity with one language over another. Critically, they also served as a useful sanity check in case we did not observe any evidence for statistical learning in the segmentation phase in the Dual Talker group. In such a scenario, the Dual Talker group could in principle still perform accurately on recognition trials, demonstrating learning of the phonology and phonotactics of the attended language (i.e., unique syllable inventories in each language), even in the absence of statistical learning of the transitional probabilities in the attended language. As with the segmentation trials, words were presented auditorily with ISI = 1,000 ms. The order of each item and the talker producing them both was alternated such that all words were heard once by each talker and were presented once in each position within test pairs.

Participants were first tested on all segmentation trials, and then on the recognition trials. The trials within each of these tasks were fully randomized. The fixed presentation order of tasks (i.e., segmentation followed by recognition) aimed to ensure (1) that segmentation performance in both the attended and unattended language was unaffected by hearing the words in isolation in recognition trials, and (2) that all participants received equal exposure to the items of both the attended and unattended language in segmentation trials before being tested on which of them occurred in the language they attended to in recognition trials.

It is important to note that both the Dual Talker and the Single Talker groups received the same segmentation and recognition trials. Consequently, the Single Talker group was also tested on segmentation trials from the unattended language, which involved words and part-words they had never been exposed to during familiarization. Further, as both the segmentation and recognition test included trials produced by either a female or a male talker, participants in the Single Talker group were familiar with only one of them at test, as the speech stream they were exposed to at familiarization was produced by either a female or a male talker. Moreover, participants in the Dual Talker group were familiar with both speaker voices, but were asked to pay attention only to either the male or the female speaker. Given that half of the test pairs were produced by the same speaker that produced the attended (or heard) language stream, while the other half were produced by the speaker that produced the unattended language stream, we included *Speaker Match* as a covariate in the statistical analysis in order to control for effects of speaker familiarity on participants performance.

#### Post-experimental debriefing

After the completion of the experiment, participants were asked questions with respect to the content and aim of the experiment as well as their impression of their selective attention performance in the familiarization phase, and their 2AFC accuracy in the testing phase. A listing of the post-experimental questions and a summary of participants’ responses to them appears in the Online Supplementary Material (OSM), Sects. 1.1 and 1.2, respectively.

## Results

### Beep-detection task

Participants demonstrated ceiling performance in the beep detection task (*M* = 11.96 beeps, *SD* = 0.2, mean RT = 839 ms, *SD* = 686 ms), indicating attentiveness during the familiarization phase.

### Segmentation task

**Fig. 2:**
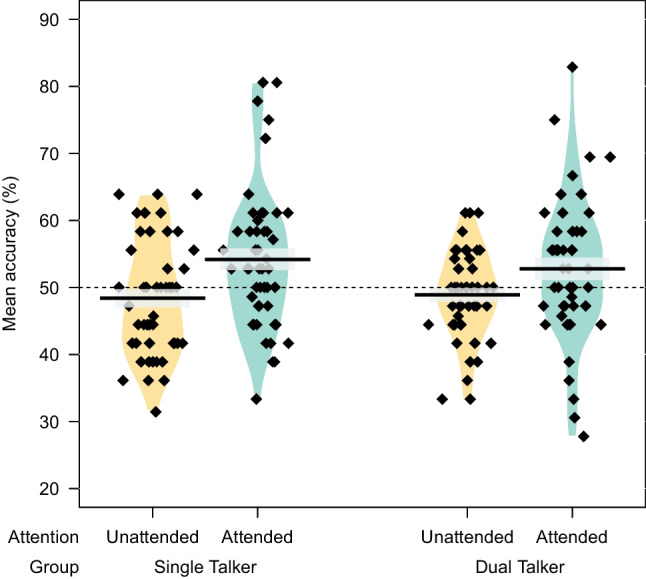
Mean accuracy for segmentation trials (word vs. part-word) from the attended and unattended language in the Single Talker (**left**) and Dual Talker group (**right**). Participants from both groups performed significantly above chance on trials from the attended language (green violins). Performance on the unattended language (never presented for the Single Talker group) was significantly worse (yellow violins) compared to the attended language. Diamonds indicate individual participants. The gray band around the mean shows SE

Segmentation trials (word vs. part-word) with missing responses due to timeout (*n* = 12; 0.17%) were excluded from the analyses. Figure 2 shows the mean and individual-participant performance for the Single and Dual Talker group.

#### Within-group segmentation performance

We ran Generalised Linear Mixed Effects Models (Quené & van den Bergh, 2008) using the ‘lme4’ package (version 1.1–26; Bates et al., 2015) in R (R Core Team, 2021) to compare segmentation performance between the attended and unattended language streams, separately for the Single and Dual Talker group. Trial accuracy was the dependent variable (correct = 1; incorrect = 0). In both analyses, the models included Test Pair as random intercepts, while the model in the Single talker group also included a by-participant random intercept and random slope for Attention. More complex random effect structures were not included due to convergence issues or model overfitting (Barr et al., 2013).

We then added *Attention* (Attended and Unattended language; dummy coded with Attended language at the reference level) as the predictor in the model (recall that the ‘unattended language’ was actually never presented in the familiarization phase of the Single talker group). We also added the covariate *Speaker Match* (deviation coded: Match = -0.5, Mismatch =  + 0.5) and its interaction with *Attention* in the analyses of both groups. Finally, we added the covariate *Lateralization* of Attended language (deviation coded: Left = -0.5, Right =  + 0.5) and its interaction with *Attention* in the Dual Talker group analysis. Full R syntax and model output is given in Tables 2 and 3.

**Table 2:** Summary of the generalized linear mixed-effects model of (log odds) accuracy scores on the segmentation test for the Single Talker group. Values for significant effects (p < 0.05) are shown in bold

Model structure	glmer(accuracy ~ 1 + Attention * Speaker Match + (1 | participant) + (1 + Attention | testpairs))				
Fixed effects	*Log-Odds*	*SE*	*CI (95%)*	*Statistic*	*p*
Intercept (Attended)	0.17	0.07	0.04 – 0.31	2.54	**0.011**
Attention (Unattended)	-0.24	0.08	-0.39 – -0.09	-3.14	**0.002**
Speaker Match	-0.01	0.10	-0.20 – 0.19	-0.08	0.939
Attention:Speaker Match	0.02	0.14	-0.26 – 0.29	0.12	0.904
Random effects	*Variance*	*SD*			
testpairs (Intercept)	0.087	0.295			
Attention (Unattended)	0.074	0.273			
Participant (Intercept)	0.047	0.218			
N _participants_	48				
N _testpairs_	72				
Observations	3450				
Marginal R^2^ / Conditional R^2^	0.004 / 0.059				

*CI* confidence interval, *SE* standard error

**Table 3:** Summary of the generalized linear mixed-effects model of (log odds) accuracy scores on the segmentation test for the Dual Talker group. Values for significant effects (p < 0.05) are shown in bold

Model structure	glmer(accuracy ~ 1 + Attention * (Speaker Match + Lateralization) + (1 | testpairs))				
Fixed effects	*Log-Odds*	*SE*	*CI (95%)*	*Statistic*	*p*
Intercept (Attended)	0.11	0.06	0.00 – 0.22	1.96	**0.049**
Attention (Unattended)	-0.15	0.07	-0.29 – -0.02	-2.22	**0.027**
Speaker Match	-0.12	0.10	-0.32 – 0.07	-1.26	0.208
Lateralization	0.23	0.10	0.04 – 0.43	2.38	**0.017**
Attention:Speaker Match	0.09	0.14	-0.19 – 0.36	0.62	0.534
Attention:Lateralization	-0.15	0.14	-0.42 – 0.12	-1.07	0.284
Random effects	*Variance*	*SD*			
testpairs (Intercept)	0.05	0.22			
N _testpairs_	72				
Observations	3378				
Marginal R^2^ / Conditional R^2^	0.005 / 0.019				

##### Single Talker group

Performance on the attended language stream was relatively low but significantly above chance (*M* = 0.54, *SE* = 0.5; Cohen’s *d* = 0.39; left green violin in Fig. 2), as shown by the intercept of the model. Importantly, we also found a significant main effect of *Attention*, with worse segmentation performance for the never-presented ‘unattended language’ (relative to attended; *M* = 0.48, *SE* = 0.5; Cohen’s* d* = 0.19; left orange violin). Note, however, the large by-participant variation, illustrated by the diamonds in Fig. 2. There was no statistically significant effect of *Speaker Match*, nor an interaction with *Attention*, suggesting participants’ performance on the segmentation task was not affected by speaker discrepancies between the test and the training phase.

##### Dual Talker group

Performance on the attended language stream was again relatively low but significantly above chance (*M* = 0.53, *SE* = 0.5; Cohen’s* d* = 0.26; right green violin in Fig. 2), as shown by the intercept of the model. There was a significant main effect of *Attention,* with participants performing worse for the unattended language (*M* = 0.49, *SE* = 0.5; Cohen’s *d* = 0.16; right orange violin) compared to the attended one. Note, however, the large by-participant variation, illustrated by the diamonds in Fig. 2. Again, the predictor *Speaker Match* and its interaction with *Attention* were not significant. Finally, there was a significant main effect of *Lateralization*, suggesting that performance was higher for participants who predominantly heard the attended language from their right side compared to those who heard it from their left. No interaction between *Lateralization* and *Attention* was found, suggesting that the effect of Lateralization held for both Attended and Unattended languages.

#### Between-group comparison of segmentation performance

An omnibus model compared performance on segmentation trials between the two groups. The omnibus included by-test pair and by-participant random intercepts, with by-test pair random slopes for *Attention* and *Group*. The by-participant random slopes for *Attention* and *Group* were removed from the random effect structure due to convergence issues (Barr et al., 2013). Fixed effects were *Attention* (dummy coded: Attended language at the reference level) and *Group* (dummy coded: Single Talker at the reference level). *Speaker match* was not included as a covariate, given the null results in the within-group analyses.

See Table 4 for model syntax and output. We found a significant intercept, demonstrating above-chance performance on the attended language in the Single Talker group. We also found a simple effect of *Attention*, with significantly worse performance in segmentation trials for the unattended language. Moreover, the predictor *Group* and its interaction with *Attention* were not statistically significant, indicating a lack of evidence for different performance between the two groups. This lack of an interaction was further assessed by performing posthoc pairwise comparisons to directly compare Single versus Dual Talker accuracy on the attended language, and also Single vs. Dual Talker group accuracy on the unattended language, using the R package ‘emmeans’ (*p*-values Bonferroni adjusted). These comparisons revealed no difference between Single vs. Dual Talker group performance on the attended language (estimate = 0.06, *SE* = 0.08, *z* = 0.749, *p* = 0.454), nor on the unattended language (estimate = -0.02, *SE* = 0.08, *z* = -0.285, *p* = 0.776).

**Table 4:** Summary of the generalized linear mixed-effects model of (log odds) accuracy scores on the segmentation test for both the Single and the Dual Talker groups. Values for significant effects (p < 0.05) are shown in bold

Model structure	glmer(accuracy ~ 1 + Attention * Group + (1 | participant) + (1 + Attention + Group | testpairs))				
Fixed effects	*Log-Odds*	*SE*	*CI (95%)*	*Statistic*	*p*
Intercept (Single-Attended)	0.17	0.07	0.04 – 0.30	2.63	**0.008**
Attention (Unattended)	-0.24	0.08	-0.39 – -0.09	-3.21	**0.001**
Group (Dual)	-0.06	0.08	-0.22 – 0.10	-0.75	0.454
Attention:Group	0.08	0.10	-0.11– 0.28	0.85	0.397
Random effects	*Variance*	*SD*			
Testpairs (Intercept)	0.10	0.31			
Participant (Intercept)	0.02	0.16			
Attention (Unattended)	0.05	0.23			
Group (Dual)	0.05	0.21			
N _participants_	95				
N _testpairs_	72				
Observations	6828				
Marginal R^2^ / Conditional R^2^	0.003 / 0.039				

*CI* confidence interval, *SE* standard error

### Recognition task

**Fig. 3:**
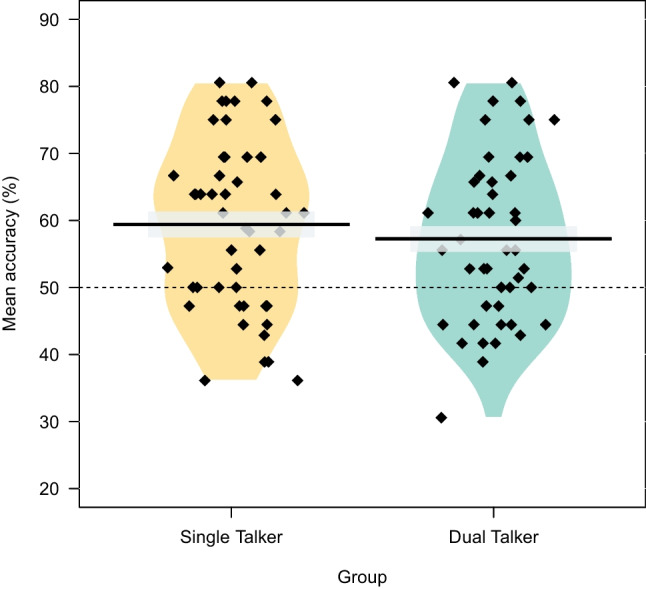
Mean accuracy on recognition trials (word from attended language vs. word from unattended language) for the Single (yellow) and Dual (green) Talker group. Performance was significantly above chance for both groups. Diamonds indicate individual participants. The gray band around the mean indicates SE

Recognition trials (i.e., a word from attended language vs. a word from unattended language) with missing responses due to timeout (*n* = 12; 0.34%) were excluded from the analyses. Figure 3 shows the mean and individual-participant performance per group.

To compare between-group performance, we incrementally built a GLMM (see Table 5) including a by-participant random intercept. Random effects for test pairs and more complex random effects for participants were not included due to model overfitting (Barr et al., 2013). Subsequently, we added *Group* (dummy coded: Single mapped onto the intercept) as a fixed effect. We also added *Speaker Match* (coded as before), and its interaction with *Group*, in order to control for effects of speaker familiarity.

**Table 5:** Summary of the GLMER for accuracy scores in the recognition test. Values for significant effects (p < 0.05) are shown in bold

Model structure	glmer(accuracy ~ 1 + Group * Speaker Match + (1 | participant))				
Fixed effects	*Log-Odds*	*SE*	*CI (95%)*	*Statistic*	*p*
Intercept (Single)	0.40	0.08	0.25 – 0.55	5.19	** < 0.001**
Group (Dual)	-0.09	0.11	-0.30 – 0.12	-0.85	0.393
Speaker Match	-0.05	0.10	-0.25 – 0.14	-0.55	0.584
Group:Speaker Match	0.06	0.14	-0.22 – 0.34	0.42	0.671
Random effects	*Variance*	*SD*			
Participant (Intercept)	0.16	0.4			
N _participants_	95				
Observations	3408				
Marginal R^2^ / Conditional R^2^	0.001 / 0.047				

The Single Talker group performed significantly above chance on the recognition task (*M* = 0.59, *SE* = 0.49; Cohen’s* d* = 0.74), as shown by the intercept. No significant effect of *Group* was observed, suggesting that the recognition performance of the Dual Talker group (*M* = 0.57, *SE* = 0.49; Cohen’s *d* = 0.58) was comparable to that of the Single Talker group. Finally, we found no significant main effect of *Speaker Match*, nor any significant interaction with *Group*.

## Discussion

Overall, the Dual Talker group demonstrated performance that was qualitatively comparable to that of the Single Talker group. This finding was also observed in an independent participant sample (*N* = 96) in an earlier Experiment S1 (see above; complete description: https://osf.io/zc543/). Specifically, both groups performed significantly above chance on segmentation trials for the attended language, providing evidence for statistical learning. However, accuracy was slightly lower (*M* = 0.54) than in some previous SL studies (e.g., 0.58 in Saffran et al., 1997), with considerable between-participant variation (cf. diamonds in Fig. 2). The large variability and modest effect size could be due to several reasons including the online format, with little control over participants’ attention, audio playback, hardware, and listening conditions; and/or multiple talkers being presented at test. The additional beep detection task, used to maintain and assess auditory attention throughout familiarization, may have also had a detrimental effect on performance (see, e.g., Franco et al., 2015). Furthermore, performance may also have been negatively affected by the inclusion of trials for the unheard/unattended language. This may have impacted participants’ perception of the task demands, or reduced their confidence for test pairs relating to the ‘attended’ language. Given these performance constraints, it is particularly remarkable that the Dual Talker group managed to achieve qualitatively comparable performance on the attended language trials as the Single Talker group, despite the addition of a competing talker.

Interestingly, we found that SL was robust against speaker match/mismatches at test versus familiarization, supporting earlier observations of generalization of SL across different voices (Estes & Lew-Williams, 2015), corroborating SL as an important mechanism in more naturalistic learning conditions. However, variability in surface form may still have had an overall reducing effect on performance, partially explaining the present modest effect size of SL. Future work should look at which of these factors critically impact the effect size of SL, and individual differences therein, especially now online testing is increasingly becoming commonplace.

Performance on segmentation trials from the unattended language was significantly worse compared to the attended language in both groups. This demonstrates an important modulating role of selective attention in SL. This is in line with the view that selective attention operates very early in perception (Bosker et al., 2020a), modulating the earliest cortical encoding of speech sounds (Mesgarani & Chang, 2012). Specifically, despite equal exposure to the two talkers, the Dual Talker group performed at chance on unattended language trials. Still, it is premature to conclude that selective attention *fully modulates* (i.e., pre-empts) SL based on this result alone because performance was modest overall, leaving little opportunity for detecting reduced yet above-chance performance. Perhaps future designs with more opportunity for learning might reveal such findings. Note, however, that the earlier Experiment S1 did induce greater overall segmentation performance in the Single Talker group yet no Group effects were observed. Still, for now, we conclude that selective attention modulates SL in multi-talker contexts, corroborating studies using non-speech streams (Daikoku & Yumoto, 2017, 2019).

We unexpectedly observed an effect of Lateralization in the segmentation data from the Dual Talker group. That is, participants who perceived the attended language as coming from their right demonstrated overall higher accuracy compared to participants who perceived the attended language as coming from their left. This observation cannot be accounted for in terms of a *right ear advantage* because the two languages were presented *to both ears*, with only ITDs and IIDs inducing a spatial segregation of the two languages. Also, this Lateralization effect did not interact with Attention, suggesting a beneficial effect of ‘paying attention to the right side’ on performance in attended *and* unattended language segmentation trials. Hence, at present, we lack an explanation for this surprising finding. Still, it does raise interesting follow-up questions such as whether SL of speech streams might be modulated by ear of presentation in dichotic listening (i.e., right ear advantage due to left hemisphere specialization for language) while SL of auditory non-speech streams would not.

Our segmentation results also show that the cognitive load experienced by hearing a language in a multi-talker context does not impact SL for the attended stream. Despite being exposed to a language in the presence of another distracting language with its own statistical regularities, the Dual Talker group was able to learn the attended language as efficiently as the Single Talker group who heard the language in a distractor-free environment (though we highlight the inter-individual variation in this regard). Thus, outcomes suggest that humans’ SL ability is relatively robust against cognitively demanding settings (Daikoku & Yumoto, 2017, 2019). This contrasts with studies suggesting that SL depreciates as cognitive load increases (e.g., Palmer & Mattys, 2016; Toro et al., 2005, 2011). This discrepancy may be due to modality differences between concurrently presented stimuli and/or to the amount of cognitive load experienced, which is hard to quantify and compare between tasks/modalities.

Altogether, our findings suggest that SL is largely maintained in ‘noisy’, and thus more naturalistic, communicative contexts. This work builds on prior demonstrations of more naturalistic SL for language, showing for example that learners can draw on the distributional properties of natural language input (Elazar et al., 2022; Pelucchi et al., 2009; Stärk et al., 2022) and that this learning generalizes across talker voices (Estes & Lew-Williams, 2015). We extend this research showing that SL can proceed in the presence of a concurrent speech stream. These findings emphasize the critical role of SL in language acquisition, and lend credence to the notion of ‘real-world’ SL more broadly—showing that the human ability to detect and draw on distributional information persists in the face of distraction, outside the confines of the conventional SL vacuum.

This observation is further supported by the recognition trial data in which participants discriminated a word from the attended language from a word from the unattended language. Both groups showed above-chance performance on the recognition trials, suggesting preferential processing and learning of the attended vs. unattended language in the Dual Talker group. Note that performance in the recognition trials was numerically higher than performance in the segmentation trials. This is likely due to the fact that performance in segmentation trials is only supported by SL of the TPs; however, performance in recognition trials is further supported by learning the languages’ phonotactics. Recall that the two languages had unique syllable inventories (/xo/ in Language A, but /xi/ in Language B). Therefore, even if someone would completely ignore the syllable TPs, they could still perform above chance on recognition trials by recognizing the unique syllable inventory. These phonotactics, together with the learned TPs, presumably contributed to the relatively higher performance on recognition trials compared to segmentation trials.

As our design used two languages with different phonotactics, listeners in the Dual Talker group were exposed to two clearly distinct ‘languages’, maximizing the phonological distance between input streams. This design was necessitated by our research question which required us to be able to separate learning from two different languages (i.e., allowing us to determine the source of learning). Still, this approach arguably reflects listening to two talkers speaking different languages. Future studies could target SL with two talkers producing the same language in order to examine whether this type of multi-talker setting perhaps enhances SL due to greater exposure to the distributional regularities. Moreover, building on the present findings about SL of adjacent TPs, future experiments could target more complex dependencies within the to-be-learned language (e.g., non-adjacent dependencies), which may be more susceptible to cognitive load than SL of adjacent dependencies (Pacton & Perruchet, 2008).

In sum, this study suggests that, when selectively listening in a multi-talker context, humans’ SL ability is relatively robust against increased cognitive load. SL is modulated by selective attention, which allows for robust segmentation performance for an attended language in a ‘cocktail party’ setting. These findings bear implications for our understanding of how language learning proceeds in more naturalistic settings, supporting the view that selective attention, a key feature of speech perception, contributes to speech segmentation and language learning.

### Supplementary Information

Below is the link to the electronic supplementary material.

Supplementary file1 (PDF 193 KB)

## Data Availability

All stimulus material, datasets, and analyses generated during this study, as well as the Online Supplementary Material, are available on the Open Science Framework: https://osf.io/7sptu/?view_only=aea95c04040e4260b88de944efcc78c6. A complete description of the earlier Experiment S1, including data, scripts, and materials, is publicly available from https://osf.io/zc543/.
