# Supplementary material for: Statistical learning at a virtual cocktail party
Source: Psychon Bull Rev. 2023 Oct 2;31(2):849–61. doi: 10.3758/s13423-023-02384-1 (PMC11061050; doi:10.3758/s13423-023-02384-1)
Supplement: Supplementary file 1 — Supplementary file1 (PDF 193 KB) [file 13423_2023_2384_MOESM1_ESM.pdf]

Supplementary Information

Statistical Learning at a Virtual Cocktail Party

Christina Papoutsi[1*], Eleni Zimianiti[1*,] Hans Rutger Bosker[1,2], Rebecca L.A. Frost[1,3]

*These two authors contributed equally to this study; order was determined by a coin toss.

[1]Max Planck Institute for Psycholinguistics, Nijmegen, the Netherlands

[2]Donders Institute for Brain, Cognition, and Behaviour, Radboud University, Nijmegen, The Netherlands

[3]Edge Hill University, United Kingdom

Corresponding author: Hans Rutger Bosker (hansrutger.bosker@mpi.nl)

PO Box 9104, 6500 HE, Nijmegen, The Netherlands

This Supplementary Information file includes:

Supplementary Text

Section 1.1. Post-experimental questions (PEQs)

Section 1.2. Summary of participants' responses to PEQs

Section 1.3. No Lateralization effect in recognition trials

Supplementary Tables

Table S1

Note that the experimental data of this study are publicly available for download from https://osf.io/7sptu/

1. Supplementary Text

1.1. Post-experimental questions

Participants in both groups (Single Talker and Dual Talker) were asked the following post-experimental questions (PEQs), three of which, namely questions 3, 5, and 7, slightly differentiate between groups:

1. Briefly describe what your task was in this experiment

2. Did you find the experiment easy or difficult? Explain.

3. (Single Talker group) The first part of the experiment consisted of the training session, where you heard an artificial language stream, and your task was to pay close attention to it. Did you manage to focus on the language during the training session? Give a rating from 0 to 100, with 0 corresponding to not being able to focus on the language throughout the entire training session, and 100 corresponding to being able to focus on the language throughout the session).

3. (Dual Talker group) The first part of the experiment consisted of the training session, where you heard two different speakers, one in your left ear and one in your right ear, and your task was to pay close attention to one of them. Did you manage to focus properly on the one speaker while ignoring the other speaker? Give a rating from 0 to 100, with 0 corresponding to not being able to focus on the one speaker (and thus ignore the other speaker) at all throughout the training session, and 100 corresponding to being able to focus on the one speaker (and thus ignore the other speaker) throughout the entire session.

4. What did you do when you were not focused on your task during the training session?

5. (Single Talker group) The second part of the experiment was the test session, where you heard two words, and your task was to identify the word that fits best to the language you heard in the training session. How ‘well’ do you think you did in the test session? Rate from 0 to 100, with 0 corresponding to not performing well at all in the test session, and 100 corresponding to performing very well.

5. (Dual Talker group) The second part of the experiment was the test session, where you heard two words, and your task was to identify the word that fits best to the language you listened to carefully in the training session. How ‘well’ do you think you did in the test session? Rate from 0 to 100, with 0 corresponding to not performing well at all in the test session, and 100 corresponding to performing very well.

6. Did you notice anything about the words in some test trials? If so, please explain.

7. (Single Talker group) During the test session, there were some trials where neither of the two words resembled the language you heard in the training session. Did you notice it? If so, what strategy did you use to make a choice?

7. (Dual Talker group) During the test session, there were some trials where neither of the two words resembled the language you paid close attention to in the training session. Did you notice it? If so, what strategy did you use to make a choice?

8. What do you think this study examines?

1.2. Summary of participants' responses to PEQs

Participants' raw individual responses (in Dutch) to each PEQ is found on OSF. In summary, participants' responses to the PEQs revealed that they understood what their tasks were during both familiarization and testing. The majority of participants found the task to focus on the speech stream during the familiarization phase (or to focus on one talker over the other for the Dual Talker group) relatively hard and boring yet doable, irrespective of the group they were assigned to. They rated their selective attention performance at an average of 82% for the Single Talker group and 68% for the Dual Talker group. With respect to the testing phase, most participants reported that they simply guessed, using their intuition to choose between the two test items. Their confidence in having performed accurately was rated at an average of 56% for the Single Talker group and 48% for the Dual Talker group.

1.3. No Lateralization effect in recognition trials

As lateralization significantly affected the Dual Talker group's performance in the segmentation test phase, a post-hoc analysis was run on the recognition test phase too, analyzing data from the Dual Talker group only. The dependent variable was trial accuracy, with Lateralization (Left and Right; dummy coded with Left at the reference level) as the predictor. The model included by-participant random intercept and random slope for Lateralization. However, the results, as shown in Table S1, indicated no significant effect of Lateralization on performance.

2. Supplementary Tables

Table S1. Summary of GLMER (log odds) for accuracy scores in the recognition test for the Dual talker group as a function of *Lateralization*.

Model structure	accuracy ~ 1 + lateralization + (1 + lateralization | Participant)				
**Fixed Effects**	*Log-Odds*	*SE*	*Conf. Int (95%)*	*Statistic*	*p*
(Intercept)	0.29	0.09	0.10 – 0.48	3.05	**0.002**
lateralization [R]	0.03	0.15	-0.27 – 0.32	0.17	0.864
**Random Effects**	*Variance*	*SD*			
Participant (intercept)	0.09	0.30			
Lateralization (Right Ear)	0.34	0.59			
N $_{\text{participants}}$	47				
Observations	1686				

Marginal R^2 / Conditional R^2	0.000 / 0.044
Observations	1686
Marginal R^2 / Conditional R^2	0.000 / 0.044
